# Osteochondroplastic tracheobronchopathy - report on 02 cases and bibliographic review

**DOI:** 10.1590/S1808-86942010000600019

**Published:** 2015-10-19

**Authors:** José Antonio Pinto, Luiz Carlos da Silva, Delmer J.P. Perfeito, Josemar dos Santos Soares

**Affiliations:** 1 Otorhinolaryngologist, Director/Head; 2 PhD in pathology - FMUSP-SP. Assistant Physician - Pathology - Medical School of Marília, FAMEMA/SP; 3 ENT. Former Resident - NOSP; 4 Occupational Medicine Physician. NOSP Resident. Núcleo de Otorrinolaringologia e Cirurgia de Cabeça e Pescoço de São Paulo/SP, Brasil (NOSP)

**Keywords:** tracheal diseases, tracheal stenosis, osteochondrodysplasia, cough

## Abstract

**Abstract:**

Osteochondroplastic tracheobronchopathy (OT) is a rare benign disorder of the lower part of the trachea and the upper part of the main bronchus characterized by numerous submucosal calcified nodules, sessile, cartilaginous and/or osseous with laryngotracheobronchial lumen projection. There are less than 400 cases reported in the word literature.

**Aim:**

to report and discuss 02 cases of OT with a bibliography review.

**Materials and Methods:**

we report on 02 cases with bibliography revision from MEDLINE, LILACS and PUBMED data.

**Study design:**

observational, descriptive, case reports.

**Conclusion:**

the symptoms result from airway obstruction, causing dry cough, dyspnea and recurrent respiratory tract infections. The diagnostic hypothesis is established by endoscopy of the upper airway (laryngo-tracheo-bronchoscopy), and the trachea/chest computed tomography is the best image exam to define tracheal nodule alterations. The differential diagnoses are papillomatosis, amyloidosis and sarcoidosis chondrosarcoma hamartoma and calcified paratracheal lymph nodes. There is no specific treatment and the prognosis is good. Surgery is restricted to moderate or severe airway obstructions. Otorhinolaryngologists must include OT in the differential diagnosis of cases of upper airway and tracheobronchial tree suggestive symptoms.

## INTRODUCTION

Osteochondroplastic tracheobronchopathy (OT) is a rare and benign disease, of unknown cause, also described as osteoplastic tracheopathy, characterized by numerous submucosal sessile nodules, cartilaginous or bony, distributed through the antero-lateral walls, projecting to the laryngotracheobronchial lumen^1^. The first publications concerning OT were made in 1855 by Wilks with the microscopic description of calcification deposits on the larynx, trachea and bronchi. Later, Dalgaard and Moersch, Rokitansky and Luschka, described similar cases in 1856 and 1857 respectively, but without microscopic proof^2^, ^3^. In 1896, von Schoroetter showed similar lesions in an indirect laryngoscopy. In 1910, Aschoff-Freiburg proposed the name osteoplastic tracheobronchopathy^4^. In the world literature it is estimated the existence of a few less than 400 cases already reported^5^, ^6^.

The nodular injuries are sessile, calcified and vary in diameter between 1-10mm. They are characterized by slow and progressive growth, they can be localized or diffuse, covered by metaplastic epithelium or normal, emerging from the perichondrium and involving all the way to the lumen of the trachea, following the route of the tracheal rings, with active hematopoietic inclusion in nodular neoformation. It may cause stenosis in the laryngotracheobronchial lumen, without involving its posterior wall, with the possibility of progressing to the main bronchi^7^.

## MATERIALS AND METHODS

In this paper we report 02 (two) cases in a descriptive observational study. All the procedures for this study were previously authorized by the Ethics in Research Committee of our Institution, under protocol # 181/09, on December 09, 2009.

## CASE PRESENTATION

### Case 1

Patient M.R.F., female, 41 years of age, complaining of intermittent dysphonia for 15 years caused by vocal strain. In 1998 she started having dyspnea at medium physical stress and, in another service, she was submitted to bronchoscopy with local biopsy, showing: “bronchial mucosa with injuries mimicking papillomatosis, located on the trachea, carina and main bronchi, with the presence of mild edema and unspecific inflammatory reaction “. In the year 2000, since her dysphonia and stress dyspnea remained, she came to our ward and her ENT exam was normal.

**Figure 1:**
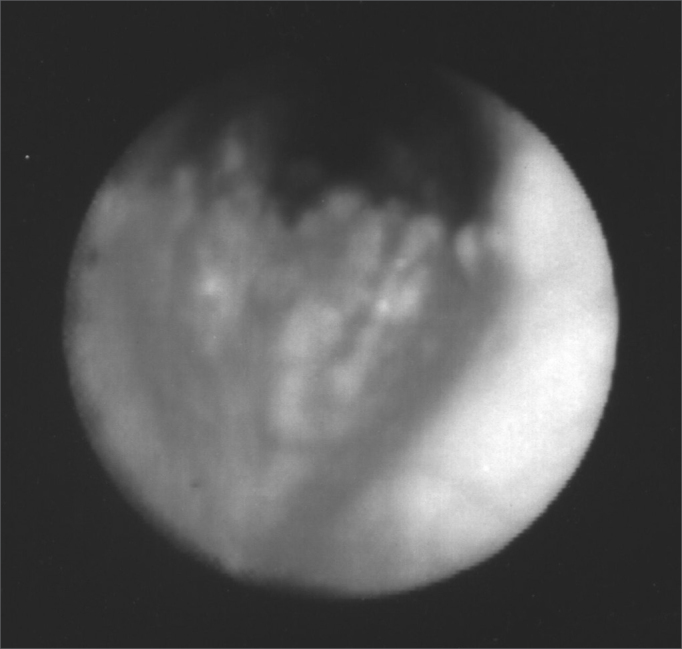
Fibro-laryngo-tracheo-bronchoscopy: subglottis, showing multiple and diffuse nodular lesions, with free vocal folds.

**Figure 2:**
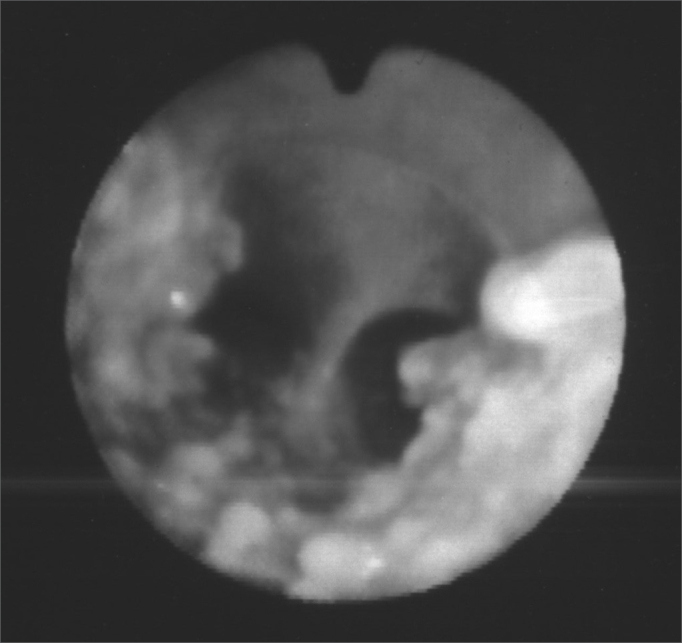
Fibro-laryngo-tracheo-bronchoscopy: showing the carina with nodular lesions, without involvement of the posterior tracheal wall.

**Figure 3:**
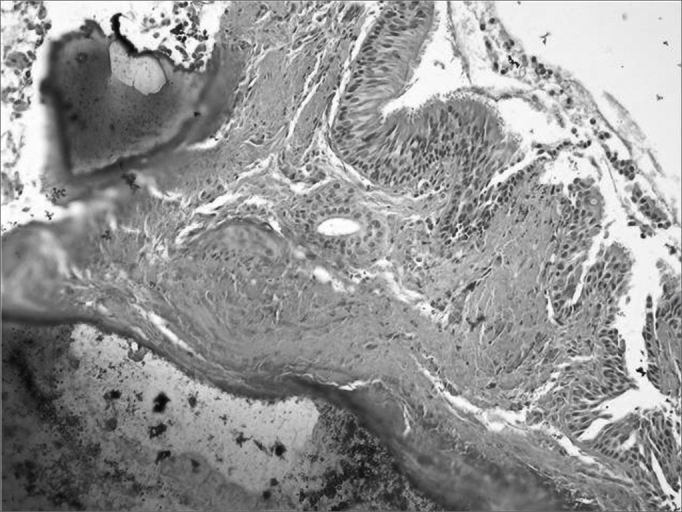
Histology slide: ossification plaques on the respiratory mucosa corion.

Upon naso-laryngo-tracheo bronchoscopy through fiber optics, she had diffuse nodular injuries on the trachea wall, on the subglottic region all the way to the carina, with a 60% reduction in the trachea lumen, without involving the posterior region (Figures 1 and 2). Upon spirometry she had mild obstructive ventilatory disorder. Her chest CT showed a thickening on the tracheal wall, with irregular nodular injuries and reduction of the tracheal lumen. In 2001, she had one episode of bronchopneumonia successfully treated with medication. In 2003, a new bronchoscopy with biopsy was carried out in order to diagnose OT (Fig. 3). Today, the patient is stable, without worsening in her clinical presentation, undergoing clinical follow up.

### Case 2

Patient A.D.B.F.C., female, 66 years old, with a history of nasal obstruction, progressive cough, hawk, night-time snoring, noisy breathing, dysphonia for 20 years. In 2002, she started having chest pain and occasional dyspnea, and a bronchoscopy she underwent showed “polypoid tumors on the anterior and lateral trachea wall all the way to the carina”. Biopsy showed a mild lymph-nuclear reaction on the submucosa, with a hyaline thickening of the basal membrane. OT was diagnosed and she was negative for amyloidosis.

**Figure 4:**
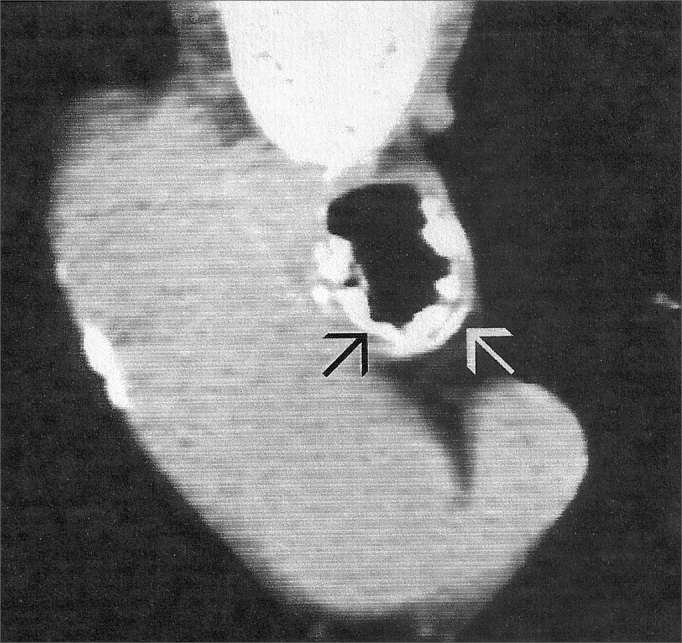
Tracheal CT scan showing osteocartilaginous nodular lesions (arrows).

Upon naso-laryngo-trachea bronchoscopy through fibroscopy, she had vocal fold edema and small trachea nodular lesions all the way to the carina. In April of 2003, she reports her dyspnea became more frequent, and a new exam showed nodular lesions in the trachea all the way to the carina, with mild lumen reduction and a chest CT scan showing thickening of the tracheal wall, with irregular nodular lesions and tracheal lumen narrowing. Today, the patient is stable, without worsening in the clinical presentation described above, only undergoing annual clinical follow up (Fig. 4).

### DISCUSSÃO

Osteochondroplastic tracheobronchopathy (OT) is a rare and benign disease, of unknown cause. It was macroscopically described by Rokitansk in 1855 and microscopically described by Wilks in 1857. Some etiopathogeny theories have been postulated: Dalgaard (1947) - states that the elastic tissue suffers a metaplasia, with cartilage formation and calcium deposition; Virchow (1863) - echondrosis and exostosis promote calcium deposition and ossification of the tracheal rings; Aschoff-Freiburg (1910) - reports changes to the tracheal elastic tissue, introducing the term osteoplastic tracheopathy and, in 1964, Secrest et al. label it osteoplastic tracheobronchopathy^8^.

Its estimated clinical incidence varies between 2-7:1,000; presenting between 25 and 85 years of age; and the 5th decade of life is the one most frequently affected. There is no gender predominance. The interval between the first symptoms and the diagnosis is about 4 years in 45% of the cases; however, it may happen after 25 years^7^. Incidental bronchoscopy findings happen to around 3:2,000-5,000. According to Secrest, it is estimated that only 5% of the cases are diagnosed during the person's life^9^.

There is no correlation with smoking, however, some studies point to an association with chronic tracheal inflammatory processes, arguing that it can be a possible factor associated with disease progression^9^, ^10^.

Its etiology and pathogenesis are unknown; however, it is believed that chronic infection, congenital anomalies, chemical or mechanical irritation, degenerative and metabolic disorders, chronic inflammation and the final stage of primary amyloidosis. Changes to the airway clearance help in disease onset after the persistent inflammatory process, resulting in repetitive infections. Bacteria such as *Klebsiella ozenae* or *Botryomicosis* have been shown in OT cases after chronic infection; however without an established correlation^8^, ^11^.

Clinically, 13-50% of the patients may remain asymptomatic. There are no pathognomonic characteristics for OT, with frequent possibilities of diagnostic mistakes and initial treatment for asthma or bronchitis, as in the cases reported. There was cough in 66% of the cases, hemoptoic cough in 60%, dyspnea in 53% and panting in 30%. The pulmonary function test is usually normal - depending on the degree of stenosis^12^, ^13^.

Chest X-Ray does not properly show the lesions, and CT scan is the image exam which best shows tracheal changes. On chest and trachea CT scans we can see the most common image changes: a few or numerous gross nodular lesions, of bony and/or cartilaginous (01-10mm) consistency, sessile and covered by an apparently normal mucosa of the trachea wall. Definitive diagnosis is confirmed through biopsy during fibro-naso-laryngotracheo-bronchoscopy^14^. In our cases we ordered chest CT scans, which showed lesions similar to the ones found in the literature.

It frequently involves the lateral and anterior walls of the distal ¾ of the trachea, progressing to both main bronchi. Its anatomical distribution happens in 42% of the trachea, 6% in the bronchi and 52% in both. There are only a handful of reports of laryngeal and subglottic involvement^9^, ^11^, ^15^. In one of the cases the lesions progressed from the subglottis and trachea to the carina. In the other, the lesions involved the lower third of the tracheal all the way to the carina.

Histologically, the mucosal bed may look normal, with areas of inflammation and necrosis, abnormal proliferative cartilaginous or bony formations on the submucosa; there may be squamous metaplasia of the columnar epithelium, calcium deposits, fragments of adipocytes, with active hematopoietic medullar bone tissue^10^, ^11^. In 2001, Leske et al. reported that in 40 biopsies they found the following changes: ossification (58%), squamous metaplasia of the respiratory epithelium (48%), cartilage (38%), calcification (20%) and amyloidosis 13%^6^, ^8^, ^14^.

There are no markers for OT. The C - reactive protein (CRP) and ESR may be altered because of the inflammatory processes. Calcium is normal and there are changes in the growth hormone and hyperphosphatemia, but it is likely a coincidence^9^. In 1996, Yokoyama et al. found an association with pulmonary cancer between 11-19%, and adenocarcinoma was the most common histological type^5^, ^12^.

In 1998, Zamani et al. found an elongated short arm of chromosome Y upon cytogenetic studies. Recent immunohistochemistry studies argue that the bone morphogenetic protein 2 (BMP-2), in high concentrations increase the expression of b-1 transformation growth factor (TGF B-1) messenger RNA (mRNA), stimulates the production of extracellular matrix proteins for chondrocytes and bone neoformation. In the normal tissue of the tracheobronchial tree the BMP-2 protein is not present^6^, ^16^. In our cases, TGF b-1 was detected in the chondrocytes and osteocytes of the submucosal nodules.

Among the differential diagnosis we find, mainly, papillomatosis, sarcoidosis, chondrosarcoma, hamartoma, amyloidosis, tuberculoid calcifications, dermatomyosites, scleroderma, Wegener's disease, sarcoidosis and calcified paratracheal lymph nodes^4^, ^7^, ^12^, ^13^.

The most common complications are: atelectasis and pneumonias^1^, ^7^.

There is no specific treatment for OT, and it is rarely necessary. In a series study carried out in 2001, Lazor et al. noticed that in 55% of the cases there was no disease progression^9^. Antibiotics are used to treat the recurrent infections of the respiratory tract. Surgery is indicated when the symptoms no longer respond to clinical treatment - tracheal segment resection, anterior laryngo-cleft, partial laryngectomy, bronchoscopic removal of the lesions. Nd:YAG laser photocoagulation and also the placement of a silicone mold can be attempted^15^, ^17^, ^18^, ^19^. Occasionally, tracheostomy is necessary. Prognosis is favorable and depends on the extension and location of the nodular lesions^8^, ^9^, ^10^, ^13^. In the cases hereby reported we decided to follow up the patients annually in the clinic.

## FINAL REMARKS

Osteochondroplastic tracheobronchopathy is a disease of unknown cause, where there is heterotopic ossification of the laryngotracheobronchial cartilage, producing symptoms directly associated with the site and degree of upper airway obstruction. Frequent treatment is unnecessary, and usually clinical control is carried out when the patient is asymptomatic or has mild symptoms. Surgical removal is restricted to moderate to severe obstructions. ENT physicians must be attentive and include OT in their list of differential diagnosis when facing symptoms suggesting upper airway and tracheal-bronchial tract.
